# Prevalence and Predictors of Violence Victimization and Violent Behavior among Youths: A Population-Based Study in Serbia

**DOI:** 10.3390/ijerph16173203

**Published:** 2019-09-02

**Authors:** Biljana Obradovic-Tomasevic, Milena Santric-Milicevic, Vladimir Vasic, Dejana Vukovic, Sandra Sipetic-Grujicic, Vesna Bjegovic-Mikanovic, Zorica Terzic-Supic, Ratko Tomasevic, Jovana Todorovic, Uros Babic

**Affiliations:** 1 Clinical Hospital Center Zemun, Faculty of Medicine, University of Belgrade, 11080 Belgrade, Serbia; 2 Institute of Social Medicine, Faculty of Medicine, University of Belgrade, 11000 Belgrade, Serbia; 3 Centre-School of Public Health and Health Management, Faculty of Medicine, University of Belgrade, 11000 Belgrade, Serbia; 4 Department of Statistics and Mathematics, Faculty of Economics, University of Belgrade, 11000 Belgrade, Serbia; 5 Institute of Epidemiology, Faculty of Medicine, University of Belgrade, 11000 Belgrade, Serbia; 6 Clinical Center of Serbia, Faculty of Medicine, University of Belgrade, 11000 Belgrade, Serbia

**Keywords:** youth, violence victimization, prevalence, socio-ecological model, health education, violence prevention

## Abstract

The study identifies the prevalence of violence victimization and the perpetration among youths, and explores the determinants and predictors using a socio-ecological model. The data of 36 variables from a representative sample of 1722 persons, ages 15–24 years, from the National Health Survey of Serbia in 2013, were analyzed by a multivariate logistic regression modeling. The study shows that 13.4% of youths experienced multi-victimization, while 10.4% were perpetrators of violence. Up to one-third of the victims were violence perpetrators. A small percentage of victims seek family and community support. Predictors of violence among youths were: male sex, households with fewer members, urban settlements, violence perpetration, self-assessed health as poor, lack of close friends and perception that it was difficult to obtain the assistance needed. Predictors of youth violence highlighted the need to improve health education, social support and community regulations, as well as strengthen the promotion of gender equality and a healthy environment.

## 1. Introduction

Violence is among the top five leading causes of death for young people aged 10–29 [1] and the elimination of violence is foreseen in the United Nations Agenda Sustainable Development Goals by 2030 [2]. Youth violence includes physical, psychological and sexual abuse, neglect, commercial or other exploitation of children (e.g., labour exploitation, forced marriage, forced criminality, domestic servitude, child soldiers), “resulting in actual or potential harm to the child’ health, survival, development or dignity in the context of establishing a relationship, responsibility, trust or power” ([3], p. 82). In the European region, over 15,000 young people aged 15–29 years die from the effects of violence, and many more are hospitalized and suffer physical, emotional, psychological or social consequences due to involvement in violence, witnessing or fear of violence [4,5].

The consequences of violence are various and often long-lasting, such as fear and anxiety, increased risk of psychiatric and behavioral problems, including suicide attempts, harmful use of tobacco, alcohol, and psychoactive substances, unsafe sex, pregnancy in adolescence and sexually transmitted disease, as well as difficulties in education, psychosocial adjustment, employment, relationships, while some victims later become perpetrators of violence [6,7,8,9,10,11]. High rates of violence reduce social security, productivity, social cohesion, destroy property in the community, and increase the cost of absenteeism, health care and justice (e.g., 1% of GDP in the USA and 2% of GDP in the countries of East Asia and the Pacific) [12]. Violence can start in early childhood, culminating in late adolescence and early adulthood [13] as young people explore their roles in the family, immediate environment and in the society. This life span is characterized by a degree of neurobiological immaturity, emotional instability, increased reactivity, and consequently inconsistent adaptability [13], which makes them susceptible to various influences.

According to the socio-ecological model [11,14], violence is a product of the biological, social, cultural and economic factors present at the individual level, the level of interpersonal relations, the community level, and at the level of the society as a whole [11]. For example, in some settings worldwide, violence is considered an acceptable way of punishing children [13], and only 10% of children enjoy the same rights to protection themselves from violence as adults [15]. In this regard, planned and controlled health education [16] that implements cognitive (educational), affective (awareness), behavioral and social systems of knowledge, attitudes and skills for individuals, at-risk groups, together with community leaders (political, religious, etc.) and institutions (schools, police, media, social and health workers, etc.) can contribute to the voluntary acceptance of healthy behavior, the elimination of violence, the promotion of youth rights, and the development of a healthy environment through building capacity building and social cohesion. A precondition for making progress in violence elimination is identifying factors that may be associated with youth violence in a particular context and which can be modified [17], as well as evaluating community resources (e.g., governmental and legal resources including police, justice, religious, school, health care and legal aids, and nongovernmental organizations, civic societies, outreach services, shelter, crises lines, peer and family support) to create programs that appear to be particularly effective in preventing youth violence [18]. The existence of comprehensive and representative estimates of the prevalence and determinants of youth violence enables policymakers and decision-makers to work on its prevention and assess the effectiveness of established activities.

Violence is unequally widespread and is likely higher in a country going through an economic, social transition, or in a post-conflict situation [11]. In Serbia, a country going through an economic transition, youths perceive violence as significant problem, along with unemployment, poverty and low education [19]. Annual National estimates show that youths (15–29 years) are victims of violent crimes in 22–28% of cases [20], commit 34–41% of all criminal acts [21] and commit over 50% of all violent crimes [21]. A high-level stakeholder from Serbia, recognized “there is no organized systemic approach to disseminating knowledge about the security risks and threats to which young people are exposed, nor the training system for acquiring skills and ability to operate in conditions of a specific security threat…and programs aimed at resolving the issue of youth safety are not in compliance with the prescribed standards” ([22], p. 33). In that regard, the aim of the study was to identify the prevalence of violence victimization and perpetration among youths in Serbia, and to explore their determinants and predictors using the socio-ecological model. The study assumptions were that more youth were multi-victimized (i.e., exposed to multiple forms of violence in multiple contexts), and while some youths were both victims and perpetrators of violence.

## 2. Materials and Methods

This is a cross-sectional study of physical and psychological violence among youths in Serbia. It is a secondary analysis of the data from the 2013 National Health Survey of the Republic of Serbia, designed and conducted by the Ministry of Health of the Republic of Serbia and the Institute of Public Health of Serbia [23]. The Institute of Public Health of Serbia gave ethical approval for the conduct of this study. A representative sample of two-stage stratified households [23] included 1722 participants aged 15 to 24 who resided in all four regions of Serbia in 2013 (response rate was 92.1% for psychological violence, 91.8% for physical violence, and from 80% to 84% for assistance requests).

The study population was persons aged 15 to 24 years who at the time of data collection lived and reside in private households in the territory of the Republic of Serbia. The National Health survey used three instruments: the household characteristics questionnaire, the face-to face interview questionnaire and the self-assessment questionnaire.

The following data were taken from the Institute of Public Health of Serbia for the purpose of this research: demographic and socio-economic characteristics of respondents, violence victimization and violence perpetration (the number of victims of physical and/or psychological violence during the previous 12 months in the family, at school/workplace or at street and the number of perpetrators of physical and/or psychological violence), also the health characteristics and risk factors of the respondents.

In this study, we measured the prevalence of the general violence victimization and perpetration. We analyzed 36 variables, including nine outcome and 27 explanatory variables (Supplementary Material Figure S1). Outcome variables were numbers of violence victimization (the number of victims of combined physical and psychological violence regardless of the place of exposure, the number of victims of physical violence regardless of the place of exposure and the number of victims of psychological violence regardless of the place of victimization), places of victimization (in a family, at school/workplace, on the street) and multi-victimization (violence in a family, at school/workplace, on the street). Explanatory variables were considered at all levels of the Socio-ecological model [11,14]. At an individual level, we analyzed gender, age, education, employment status, wealth index, the self-assessed health, chronic diseases, tobacco smoking, alcohol binge-drinking, i.e., having six or more drinks in a row during an event, and the same-sex intercourse. We explored the close relationships, including marital status, the number of close friends, and members in the households. At the community level, we studied the type of settlement and region. At the social level, we examined how respondents perceive neighbors’ interest in them (in their life, in what is happening to them), whether they perceive difficulty obtaining the necessary assistance, whether they have asked for help from relatives, police, teachers, social workers, health workers, and through Help line in a case of violence, and whether respondents followed health information through conventional media such as television, internet, print media, and radio.

The Kolmogorov-Smirnov test examined the normal distribution of data. For continuous variables that follow a normal distribution, the data are presented as the mean and standard deviation (number of household members). For categorical variables, data are displayed as absolute and relative frequencies. Pearson Chi-squared test and one-factor ANOVA were used to test for statistically significant difference at *p* < 0.05. Multiple logistic regression was used to identify predictors of violence among significant explanatory variables, (all statistically significant variables with *p* < 0.05 were included, and some with low frequency among categories were re-coded into single e.g., bad/very bad, good/very good, etc.), except tobacco smoking, alcohol binge drinking, and same-sex intercourse, due to low response rate (25%, 51% and 50%, respectively). To determine the strength of predictors for different types of violence, a 95% confidence interval was calculated by using the odds ratio (OR). All statistical analyses were performed using the SPSS, version 22.0 [24].

## 3. Results

### 3.1. Characteristics of Youths (15–24 Years Age) in Serbia in 2013

In the representative sample of 1722 (100%) young people, 51% were females and 60% were in the age interval of 15–19 years (Supplementary Material Table S1). The majority of respondents were from urban settlements (56%) and from the region of Sumadija and Western Serbia (30%). The percentage of households in all quintiles of wealth index ranged from 18% to 22%, and each household consisted of 4.4±1.6 persons. Less than 5% of respondents had completed higher education, less than 10% were married and around 12% were employed. They self-assessed their health as good/very good (95%), and about 7% had a chronic disease. On average, the respondent had 3.0 ± 0.7 close persons, and most of them thought that neighbors have interest in them (93.4%) and that they could easily get assistance from the neighbors when necessary (87.5%). However, they rarely follow health information via conventional media (e.g., only half of them watched television or used the Internet) (Supplementary Material Table S1).

### 3.2. Violence Victimization Among Youths (15–24 Years Age) in Serbia in 2013

The study showed that 13.4% of young people were victims (mostly in the family and on the street) of combined psychological and physical violence (2.8% and 7.3%, respectively), while 5.3% were victims of psychological and 1.3% of physical violence (Supplementary Material Table S2). Also, 6.4% of respondents were victims of psychological violence at school/workplace, while 4.1% were victims of physical violence on the street. Youths who were and those who were not victims of combined type of violence significantly differed (*p* < 0.05) regarding individual characteristics such as sex, violence perpetration, self-assessed health status, binge-drinking and sexual relationship with person of the same sex, close relationship characteristics such as number of members in the household, and number of close friends, community characteristics such as type of residence, and social characteristics such as perceived difficulty in getting necessary assistance (Table 1). The prevalence of combined violence victimization was 11.1% for females and 15.5% for males, 15.5% for youths from urban settlements and 10.7% for those from other settlements, among respondents with 2 or less close friends it was 18.2%, while among respondents with 3 or more friends it was 11.8%, among those who found it very difficult or difficult to obtain the necessary help it was 19.2%, while among their counterparts it was 12.6%.

A greater psychological violence victimization was found among respondents who rated their health as bad/very bad (14.3%), with chronic disease (11.4%), lived in urban settlements (7.8%), with two or less close friends (7.5%), and among those who thought that neighbors had little or no interest in them (7.4%), than among their counterparts (Supplementary Material Table S3). Larger physical violence victimization was among respondents with no close friends (16.7%), who rated their health as bad/very poor (14.3%), males (2.2%), and from rich/the richest households (1.9%) than among their comparators (Supplementary Material Table S4).

Greater domestic violence victimization was more likely among psychologically and physically violence perpetrators (about 10.5 times and 6.4 times, respectively), those who rated their health as bad/very bad (10 times), with chronic disease (three times), and among those with two or less close friends and among those who thought that people had an average, little or no interest in them, than among their counterparts (Supplementary Material Table S5). The higher violence victimization at school/workplace was among the respondents who rated their health as bad/very bad (25%), and in primary school (9.6%), than among their comparators (Supplementary Material Table S6). The prevalence of street violence victimization was higher in urban settlements (9.4%) and among respondents from the rich/richest households (8.4%), than among their counterparts (Supplementary Material Table S7). Respondents who practice the same-sex intercourse were more often victims of domestic violence (Supplementary Material Table S5) while those who practiced binge-drinking were more often victims of street violence (Supplementary Material Table S7).

About two-fifths of all respondents who were victims of violence asked for help from their relatives or friends, while significantly fewer victims contacted the police or teachers, professors, social workers, health workers or SOS service for that reason (Supplementary Material Table S11).

### 3.3. Predictors of Violence Victimization among Youths (15–24 Years Age) in Serbia in 2013

Respondents who assessed their own health as bad/very bad, and physically or psychologically violent perpetrators were more likely to be exposed to combined violence than their counterparts (5.4 times, and 7.3 times or 3.3 times, respectively) (Table 2). Those who were psychologically violent perpetrators had 8.4 times more likelihood for psychological violence victimization than their comparators. Respondents who assessed their own health as bad/very bad, physically violent perpetrators and males were more likely to be victims of physical violence than their comparators (235.5 times, 15.8 times and 13.6 times, respectively) (Table 2). Respondents with 1–2 friends and those with three or more friends were less likely to be victims of physical violence than those without close friends (92% and 95%, respectively) (Table 2).

Respondents who have assessed their own health as bad/very bad, from urban settlements, and males were more likely to be victims of street violence (6.5 times, 2.5 times and 2.4 times, respectively) (Table 3). Psychologically violent perpetrators were more likely exposed to be victims, domestic violence, school/workplace and street violence (5.3-times, 7.3-times, and 7.0-times, respectively), while physically violent perpetrators were more likely to be victims of violence at school/workplace and on the street (3.0 times and 3.0 times respectively) (Table 3).

### 3.4. Violence Perpetration among Youths (15–24 Years Age) in Serbia in 2013

According to the study, 10.3% of respondents were perpetrators of psychological and physical violence (7% psychological and 7.1% physical) (Supplementary Material Table S12). Young people who were not psychologically and physically violent perpetrators differed significantly (*p* < 0.05) in terms of individual characteristics including sex, self-perception of their health status, practicing binge-drinking and same-sex intercourse, and close relationship status such as marital status and number of household members (Supplementary Material Table S8). Combined violence perpetration was more prevalent among those who self-assessed their health as bad/very bad (35.7%), males (15.9%), single persons (12.0%), and youths from households with more members, than among their counterparts (Supplementary Material Table S8).

Respondents who were and those who were not psychologically violent perpetrators significantly differed (*p* < 0.05) in terms of individual characteristics including sex, household wealth index, self-perception of their health status, and chronic disease, family status, and close relationship status such as number of household members and marital status (Supplementary Material Table S9). Psychologically violence perpetration was more prevalent among respondents who were also physically violent perpetrators (46.5%), who self-assessed their health as bad/very bad (35.7%), chronically ill (11.7%), youths from the richest households (10.8%), males (8.9%), and single persons (7.4%), than among their counterparts (Supplementary Material Table S9). The prevalence of only psychological violence perpetration was lower in households with more family members (Supplementary Material Table S9).

Respondents who were perpetrators of physically violence and those who were not, differed significantly (*p* < 0.05) in terms of individual characteristics including sex, psychological violence perpetration, binge-drinking, then, close relationship status such as family status, and social characteristics such as perceptions of difficulties in obtaining the necessary assistance (Supplementary Material Table S10). Physical violence perpetration was more common among psychologically violent perpetrators (46.9%), men (11.8%), those who felt that obtaining necessary help was difficult and very difficult (11.7%) and single persons (7.6%), than among their counterparts (Supplementary Material Table S10).

### 3.5. Predictors of Violence Perpetration among Youths (15–24 Years Age) in Serbia in 2013

Male respondents were physically and psychologically more likely to be violent (2.5 times), also those who have assessed their health as average or bad/very bad (2.2 or 5.5 times respectively), than their counterparts (Table 4). Physically violent perpetrators had greater likelihood for psychologically violence perpetration (25.6 times), as well as those who have assessed their health as average or bad/very bad and (2.2 times or 5.5 times, respectively), than their counterparts, while 93% less likelihood for it had youths from households with more members (Table 4). More likely to be physically violent were psychologically violent perpetrators (21.5 times), male respondents (4.5 times), and those who perceived difficult/very difficult getting necessary help when exposed to violence (1.9 times), than their counterparts (Table 4).

## 4. Discussion

In Serbia, the violence victimization was greater than the prevalence of the violence perpetration (13.4% vs. 10.3%). The prevalence of violence victimization is in the range of median prevalence estimated in some countries (from 5% in the Czech Republic to 37% in Australia) [25]. This prevalence for young males (15.8%) is similar to the prevalence found worldwide, from 5% to 15% [3], and is below 30%, as reported mainly in African and eastern Mediterranean countries [26]. The prevalence among young women in Serbia (11.1%) is much higher than in other countries, where this prevalence ranges from 3% up to 5% [3]. The violence victimization in Serbia varies over time; it is higher than in 2000 (9%) but lower than in 2006 (14%). There has been a decrease in physical violence victimization (from 6.0% in 2006 to 1.3% in 2013) [27], suggesting that multisectoral prevention of physical violence was somewhat effective [22]. From 2006 to 2013, the violence perpetration increased slightly from 10% [28] to 10.3%, mainly among persons aged 15 to 19 years (from 8% to 8.5%). Up to one-third of victims were also perpetrators of violence.

Young people were exposed to more than one type and place of violence. Researchers have shown that 71% of children aged 2–17 years reported at least one victimization, 67% two incidents, 25% three separate incidents of violence, and 22% at least four incidents [29]. As in the eight countries of Eastern Europe (Albania, Latvia, Lithuania, Montenegro, Romania, the Russian Federation, the Republic of Macedonia and Turkey) [30], the frequency of psychological violence is greater than physical violence among young people aged 18 to 25 years. Our study showed that the highest percentage of respondents were victims of physical violence in the street, while psychological violence was more common at school/work and in the family environment. However, only less than one-fifth of these victims reported and requested family and police support, implicitly suggesting that the official sub-registration is likely to be large. Precisely, the study shows that number of victims is likely to be higher: three times more than reported in the family, eight times more than registered by police officers or at schools/work, 21 times more than reported by social welfare services, 26 times more than registered by health services and 35 times more than recorded by the Help line. The situation is similar in high-income countries, where only 0.3 to 10 per cent of victims among children have reported maltreatment [31,32]. The reasons for underreporting might be lack of or fragmentation of services that assist victims of violence, their concentration in major cities only, inadequate capacities that are not upgraded, or poor implementation of anti-violence laws [3].

In Serbia, as well as worldwide [33], similar factors apply to youth violence, indicating that each country could use them to significantly improve prevention [34]. In this study, youth violence victimization was associated with many individual characteristics (such as male sex, adolescent period, affluent households, self-assessed health as poor, chronic diseases, violence perpetration, binge-drinking, and same-sex relationships), characteristics of close relationship (less-member households and lack of close friends or partners), community (urban settlement) and society, (the perception that neighbors have no interest in them and find it difficult to get practical help). Predictors of violence victimization were male gender, violent perpetration, and self-assessed health as poor, lack of close friends and urban settlements. Predictors of violence perpetration were male gender, self-assessed health as average or poor, less-member households and the perception that assistance is difficult to obtain when needed.

The study showed that Socio-ecological model is useful for analyzing youth violence, as it provided evidence that victims also act violently and that equal attention should be paid to the health education of victims as well as perpetrators, in order to end the vicious circle of violence. Socio-cultural theories explain that aggressive behavior is primarily a product of a cultural and social structure in which widespread social inequalities, lack of opportunities for development, including unemployment and delinquency, often present in post-conflict situations and crisis, can contribute to creating a subculture of violence in society [5,35]. This study confirms that most characteristics of all levels of the Socioecological model are relevant explanators of violence victimization and perpetration, and need attention in programs for violence prevention. People resort to violence when they feel deprived of or have limited access to achieving desired goals through legitimate channels [36], while in our study, some respondents perceived a lack of support from community and closest friends. The fact that only two-fifths of violence victims sought help from relatives or friends, while much less from community services, suggests that as in other countries after the civil strife [5], the level of social cohesion in Serbia is low, while gender norms are likely to be tolerant of violence perpetration [37]. Unlike other communities [5], in Serbia, the prevalence of violence victimization in Serbia is higher in households with fewer members and with the highest wealth index compared to other households. The relationship between residence type and violence is seen complex; we found more common youth violence in urban settings than in other areas, probably because of much more opportunities to access alcohol (in bars and nightclubs) and the promotion of free alcoholic beverages (i.e., binge-drinking) and alcopops [38,39], despite resources and services to prevent violence are concentrated in cities. This finding supports the implementation of measures to reduce the availability of alcohol to young people, such as increasing prices and prohibiting the sale of alcohol to minors, reducing advertising and promoting alcohol, and limiting the number of shops selling alcohol and tobacco [40].

The strength of the study comes from a representative sample of the young population, a high level of expertise in instrument design, a high response rate, and a comprehensive approach that encompasses 36 variables on the four levels of the Socio-ecological model and outweighs the potential biases often seen in surveys [26]. A cross-sectional study design allowed examination of predictors rather than causes of youth violence in Serbia. Because the sample did not include young people in institutional care, homeless, or in Kosovo and Metohija, and the instrument did not cover other relevant factors such as knowledge and attitudes towards violence, the study findings could not be generalized. Future studies should explore these aspects of youth violence, availability and influence of community resources to prevent violence as well as reasons for violence underreporting, and individual, peer, family and social protective factors (e.g., commitment to school, involvement in prosocial activities, behavior rules, religious beliefs, engagement of parents and teachers, shared activities with parents, social skills/competencies, provision of models of constructive coping). An additional value of the study is evidence that policy-makers can use to strengthen social and community capacities. The good examples of violence prevention methods such as health education, building social capital and practical assistance against violence through mentoring and peer education programs, strategic communication, promotion of gender equality and attitudes, regulations and behaviors that reduce binge-drinking and other social mechanisms [18,41,42] should be considered in Serbia.

## 5. Conclusions

Overall, the increased prevalence of violence perpetration among young people in Serbia, especially the women victimization, suggests the need to strengthen the efforts for violence reduction in society. Determinants and predictors of psychological and physical violence have highlighted the necessity to improve a range of programs aimed at the eliminating violence, also promoting health education at the individual, relationship, community and social level, as well as developing healthy and violence-free environments. An effective health education network can raise awareness of the importance of empowering young people to solve problems and take control of their behavior, maintain healthy relationships, and become more informed about the work of institutions, programs, procedures and regulations to prevent violence.

## Figures and Tables

**Table 1:** Distribution of respondents according to combined (psychological and physical) violence victimization regardless to their demographic and other characteristics, young people in Serbia age 15–24 years (n = 1546), 2013.

Respondents’ Characteristics	Combined (Psychological and Physical) Violence Victims				
	No		Yes		*p* *
	n = 1339		n = 207		
	n	%	n	%	
Individual level					
Sex					0.007
Female	705	52.7	88	42.5	
Male	634	47.3	119	57.5	
Age					0.054
15–19	792	59.1	137	66.2	
20–24	547	40.9	70	33.8	
Employment status					0.766
Unemployed	1174	87.7	183	88.4	
Employed	165	12.3	24	11,6	
Wealth index					0.080
1st (the lowest)	237	17.7	35	16.9	
2nd	267	19.9	40	19.3	
3rd	264	19.7	39	18.8	
4th	297	22.2	34	16.4	
5th (the highest)	274	20.5	59	28.5	
Education level					0.485
Primary or lower	517	38.7	89	43.0	
Secondary	760	56.8	109	52.7	
Tertiary	62	4.6	9	4.3	
Perpetrate psychological violence					<0.001
No	1275	96.7	129	66.8	
Yes	43	3.3	64	33.2	
Perpetrate physical violence					<0.001
No	1323	4.1	139	72.8	
Yes	56	95.9	52	27.2	
Own health self-perception					<0.001
Very good	877	65.5	110	53.1	
Good	408	30.5	78	37.7	
Average	48	3.6	12	5.8	
Bad	5	0.4	5	2.4	
Very bad	1	0.1	2	1.0	
Chronic disease					0.060
Yes	93	38.6	22	10.6	
No	1246	56.8	185	89.4	
Tobacco smoking	396		91		0.447
Yes	232	58.6	75	82.4	
No	84	21.2	16	17.6	
Binge drinking **	681	100	151	100	0.019
Yes	418	61.4	43	28.5	
No	263	38.6	108	71.5	
Same-sex intercourse					0.048
Yes	20	2.9	8	6.5	
No	658	97.1	115	93.5	
Close-relationship level					
Marital status					0.599
Single	1207	90.1	189	91.3	
Married	132	9,9	18	8,7	
Number of close friends (to turn to in case of a serious personal problem)					0,002
None	6	0.4	4	1.9	
1 or 2	308	23.0	66	31.9	
3–5	724	54.1	99	47.8	
6 or more	301	22.5	38	18.4	
Number of household members arithmetic mean ± standard deviation (range of arithmetic mean) minimum-maximum	4.45 ± 1.620		4.15 ± 1.462		0.013#
	(4.36–4.53)		(3.95–4.35)		
	1–15		1–9		
Community level					
Type of settlement					0.006
Other settlements	603	45.0	72	34.8	
Urban settlement	736	55.0	135	65.2	
Region					0.433
Vojvodina	314	23.5	50	24.2	
Belgrade	287	21.4	45	21.7	
Sumadija and Western Serbia	404	30.2	52	25.1	
Southern and Eastern Serbia	334	24.9	60	29.0	
Societal level					
Neighbors’ interest in their life, in what is happening to them					0.066
Very interested	828	61.8	113	54.6	
Interested	426	31.8	76	36.7	
Indifferent	59	4.4	10	4.8	
Little interested	22	1.6	5	2.4	
Not interested	4	0.3	3	1.4	
Getting necessary help from neighbor is					0.037
Very easy	246	18.4	30	14.5	
Easy	514	38.4	66	31.9	
Possible	427	31.9	75	36.2	
Difficult	123	9.2	28	13.5	
Very difficult	29	2.2	8	3.9	
Tracking health information by media					
TV (yes/occasionally)	771	57.9	115	56.1	0.572
No	561	42.1	90	43.9	
Internet (yes/occasionally)	746	56.0	119	58.0	0.400
No	586	44.0	86	42.0	
Newspapers (yes/occasionally)	295	26.1	75	36.6	0.962
No	836	73.9	130	63.4	
Radio (yes/occasionally)	263	19.7	39	19.0	0.800
No	1069	80.3	166	81.0	

* Pearson Chi Square test. ** Consuming six or more alcoholic beverages in a row. # one-way ANOVA F = 6173; df = 1.

**Table 2:** Determinants and predictors of violence victimization (Odds Ratios and 95% Confidence Interval) by type of violence, youth (15–24 years) in Serbia, 2013.

Respondents’ Characteristics (1 = Reference)	Combined Psychological and Physical Violence (n = 1546, Response Rate 90%)	Psychological Violence (n = 1448, Response Rate 84%)	Physical Violence (n = 1357, Response Rate 79%)
Sex			
Females	1	1	1
Males	1.50 (1.12–2.02) *p* = 0.007	0.98 (0.66–1.45) *p* = 0.978	4.26 (1.72–10.54) *p* = 0.002
Marital status			
Single	1	1	1
Married	0.87 (0.52–1.46) *p* = 0.599	1.03 (0.54–1.96) *p* = 0.937	0.68 (0.16–2.88) *p* = 0.598
Type of settlement			
Other settlements	1	1	1
Urban settlement	1.54 (1.13–2.08) *p* = 0.006	1.53 (1.02–2.30) *p* = 0.041	1.82 (0.82–4.03) *p* = 0.139
Number of household members			
	0.88 (0.80–0.97) *p* = 0.013	0.88 (0.77–1.00) *p* = 0.054	1.82 (0.82–4.03) *p* = 0.139
Wealth Index			
1st quintile (the lowest)	1	1	1
2nd quintile	1.01 (0.62–1.95) *p* = 0.954	0.89 (0.44–1.78) *p* = 0.737	1.77 (0.44–7.18) *p* = 0.421
3rd quintile	1.00 (0.61–1.63) *p =* 0.999	1.06 (0.54–2.06) *p* = 0.837	2.39 (0.63–9.13) *p* = 0.201
4th quintile	0.77 (0.47–1.28) *p* = 0.320	1.08 (0.56–2.07) *p* = 0.817	0.532 (0.088–3.21) *p* = 0.491
5th quintile (the highest)	1.46 (0.93–2.29) *p* = 0.103	1.63 (0.88–3.01) *p* = 0.119	2.88 (0.78–10.60) *p* = 0.111
Education level			
Elementary	1	1	1
Secondary	0.83 (0.62–1.13) *p* = 0.235	1.18 (0.78–1.79) *p* = 0.430	0.63 (0.29–1.36) *p* = 0.238
Tertiary	0.84 (0.41–1.76) *p* = 0.649	1.10 (0.42–2.89) *p* = 0.859	1.19 (0.26–5.37) *p* = 0.820
Age years			
15–19	1	1	1
20–24	0.76 (0.57–1.02) *p* = 0.065	0.91 (0.62–1.35) *p* = 0.654	0.51 (0.23–1.10) *p* = 0.087
Own health self-perception			
Good/ very good	1	1	1
Average	1.71 (0.89–3.28) *p* = 0.107	2.43 (1.16–5.11) *p* = 0.019	1.00 (0.89–1.21) *p* = 0.998
Bad/very bad	7.97 (2.65–23.98 *p* < 0.001)	2.16 (0.26–18–15) *p* = 0.477	7.65 (0.89–65.66) *p* = 0.064
Chronic disease			
No	1	1	1
Yes	2.31 (1.62–3.28) *p* < 0.001	2.99 (1.93–4.63) *p* < 0.001	1.10 (0.38–3.20) *p* = 0.861
Perpetrate psychological violence			
No	1	1	1
Yes	14.71 (9.60–22.54) *p* < 0.001	10.9 (6.44–18.5) *p* < 0.001	14.83 (6.30–34.89) *p* < 0.001
Perpetrate physical violence			
No	1	1	1
Yes	8.46 (5.58–12.83) *p* < 0.001	3.60 (1.93–6.72) *p* < 0.001	16.53 (7.29–37.78) *p* < 0.001
Number of close friends			
None	1	1	1
1–2	0.32 (0.09–1.17) *p* = 0.085	0.19 (0.05–0.82) *p* = 0.026	0.17 (0.02–1.61) *p* = 0.124
3 or more	0.20 (0.06–0.72) *p* = 0.014	0.15 (0.04–0.61) *p* = 0.008	0.11 (0.01–0.97) *p* = 0.047
Neighbors’ interest for them			
Interested/very interested	1	1	1
Indifferent	1.12 (0.57–2.24) *p* = 0.738	1.97 (0.95–4.10) *p* = 0.069	1.00 *p* = 0.997
Not interested at all/less interested	2.04 (0.91–4.58) *p* = 0.083	1.49 (0.44–5.02) *p* = 0.518	1.00 *p* = 0.998
Getting the necessary help			
Easy/very easy	1	1	1
Average	1.39 (1.01–1.92) *p* = 0.046	0.70 (0.38–1.30) *p* = 0.259	3.12 (1.30–7.49) *p* = 0.011
Difficult/very difficult	1.87 (1.23–2.86) *p* = 0.003	1.17 (0.63–2.19) *p* = 0.624	4.37 (1.56–12.25) *p* = 0.005

**Table 3:** Determinants and predictors for violence victimization (Odds Ratios and 95% Confidence Interval) by place, youth (15–24 years) in Serbia, 2013.

Respondents’ Characteristics (1 = Reference)	Family (n = 1512, Response Rate 87.8%)	School/Workplace (n = 1484, Response Rate 86.2%)	Street (n = 1493, Response Rate 86.7%)
Sex			
Females	1	1	1
Males	0.94 (0.52–1.71) *p* = 0.834	1.36 (0.91–2.02) *p* = 0.130	2.69 (1.77–4.09) *p* < 0.001
Type of settlement			
Other settlements	1	1	1
Urban settlement	1.69 (0.89–3.21) *p* = 0.110	1.12 (0.75–1.66) *p* = 0.594	2.23 (1.45–3.43) *p* < 0.001
Number of household members			
	0.88 (0.72–1.08) *p* = 0.212	0.90 (0.79–1.03) *p* = 0.125	0.86 (0.76–0.98) *p* = 0.027
Wealth index			
1st quintile (the lowest)	1	1	1
2nd quintile	0.46 (0.18–1.17) *p* = 0.461	1.62 (0.83–3.19) *p* = 0.160	0.97 (0.49–1.91) *p* = 0.933
3rd quintile	0.40 (0.15–1.07) *p* = 0.068	1.45 (0.73–2.90)*P* = 0.292	1.04 (0.53–2.03) *p* = 0.906
4th quintile	0.48 (0.20–1.19) *p* = 0.114	0.99 (0.48–2.04) *p* = 0.970	074 (0.37–1.49) *p* = 0.400
5th quintile (the highest)	0.61 (0.26–1.41) *p* = 0.247	1.67 (0.86–3.25) *p* = 0.128	1.99 (1.10–3.60) *p* = 0.023
Education level			
Elementary	1	1	1
Secondary	0.72 (0.39–1.34) *p* = 0.299	053 (0.35–0.79) *p* = 0.002	1.30 (0.86–1.97) *p* = 0.210
Tertiary	1.29 (0.37–4.46) *p* = 0.686	0.58 (0.20–1.65) *p* = 0.309	1.40 (0.57–3.46) *p* = 0.460
Age years			
15–19	1	1	1
20–24	1.31 (0.71–2.40) *p* = 0.381	0.52 (0.34–0.78) *p* = 0.002	1.03 (0.70–1.52) *p* = 0.873
Own health self-perception			
Good/ very good	1	1	1
Average	3.67 (1.38–9.72) *p* = 0.009	0.47 (0.11–1.94) *p* = 0.294	2.10 (0.97–4.55) *p* = 0.059
Bad/very bad	11.67 (3.08–44.20) *p* < 0.001	4.43 (1.18–16.62) *p* = 0.027	9.76 (3.04–31.3) *p* < 0.001
Chronic disease			
No	1	1	1
Yes	1.60 (0.76–3.37) *p* = 0.220	2.46 (1.56–3.87) *p* < 0.001	2.27 (1.45–3.56) *p* < 0.001
Perpetrate psychological violence			
No	1	1	1
Yes	9.05 (4.56–17.94) *p* < 0.001	12.84 (7.90–20.9) *p* < 0.001	16.15 (10.09–25.9) *p* < 0.001
Perpetrate physical violence			
No	1	1	1
Yes	6.46 (3.19–13.11) *p* < 0.001	8.51 (5.26–13.77) *p* < 0.001	10.0 (6.24–16.13) *p* < 0.001
Number of close friends			
None	1	1	1
1–2	0.21 (0.04–1.07) *p* = 0.211	1.00 *p* = 0.999	0.24 (0.06–0.96) *p* = 0.042
3 or more	0.09 (0.02–0.43) *p* = 0.003	1.00 *p* = 0.999	0.16 (0.04–0.65) *p* = 0.01
Neighbors’ interest in them			
Interested/very interested	1	1	1
Indifferent	3.06 (1.16–8.07) *p* = 0.024	0.82 (0.29–2.30) *p* = 0.709	0.81 (0.29–2.27) *p* = 0.809
Not interested at all/less interested	5.60 (1.86–16.81) *p* = 0.002	0.91 (0.21–3.86) *p* = 0.895	2.99 (1.20–7.73) *p* = 0.018
Getting the necessary help			
Easy/very easy	1	1	1
Average	1.45 (0.74–2.85) *p* = 0.279	1.21 (0.79–1.86) *p* = 0.386	1.30 (0.83–2.03) *p* = 0.254
Difficult/very difficult	2.21 (0.98–4.96) *p* = 0.055	1.21 (0.66–2.23) *p* = 0.541	2.93 (1.78–4.82) *p* < 0.001

**Table 4:** Determinants and predictors for violence perpetration (Odds Ratios and 95% Confidence Interval), youth (15–24 years) in Serbia, 2013.

Respondents’ Characteristics (1 = Reference)	Psychological and Physical (n = 1574, Response Rate 91.4%)	Psychological (n = 1548, Response Rate 89.9%)	Physical (n = 1548, Response Rate 89.9%)
Sex			
Females	1	1	1
Males	2.59 (1.85–3.62) *p* < 0.001	1.76 (1.19–2.61) *p* = 0.005	4.99 (3.07–8.11) *p* < 0.001
Type of settlement			
Other settlements	1	1	1
Urban settlement	1.29 (0.94–1.78) *p* = 0.115	1.40 (0.94–2.09 < 9 *p* = 0.098	1.18 (0.80–1.74) *p* = 0.416
Marital status			
Single	1	1	1
Married	0.35 (0.16–077) *p* = 0.008	0.41 (0.16–1.01) *p* = 0.054	0.33 (0.12–0.90) *p* = 0.031
Number of household members			
	0.91 (0.82–1.00) *p* = 0.059	0.77 (0.67–0.89) *p* < 0.001	1.01 (0.90–1.14) *p* = 0.82
Wealth index			
1st quintile (the lowest)	1	1	1
2nd quintile	0.97 (0.57–1.65) *p* = 0.916	0.91 (0.46–1.76) *p* = 0.769	0.98 (0.50–1.93) *p* = 0.982
3id quintile	0.91 (0.53–1.56) *p* = 0.772	0.78 (0.39–1.56) *p* = 0.478	1.19 (0.62–2.29) *p* = 0.604
4th quintile	0.99 (0.59–1.67) *p* = 0.972	0.97 (0.51–1.86) *p* = 0.923	0.94 (0.48–1.84) *p* = 0.852
5th quintile (the highest)	1.60 (0.99–2.59) *p* = 0.057	1.74 (0.97–3.13) *p* = 0.064	1.76 (0.96–3.21) *p* = 0.067
Education level			
Elementary	1	1	1
Secondary	0.88 (0.64–1.21) *p* = 0.423	0.67 (0.45–1.00) *p* = 0.048	1.15 (0.76–1.73) *p* = 0.504
Tertiary	0.88 (0.41–1.92) *p* = 0.756	0.75 (0.29–1.94) *p* = 0.552	1.26 (0.52–3.08) *p* = 0.613
Age years			
15–19	1	1	1
20–24	0.73 (0.53–1.00) *p* = 0.053	0.62 (0.42–0.92) *p* = 0.018	0.87 (060–1.29) *p* = 0.497
Own health self-perception			
Good/very good	1	1	1
Average	1.79 (0.92–3.51) *p* = 0.089	2.18 (1.01–4.71) *p* = 0.048	0.93 (0.33–2.61) *p* = 0.890
Bad/very bad	4.61 (1.53–13.93) *p* = 0.007	8.01 (2.63–24.36) *p* < 0.001	3.61 (0.99–13.15) *p* = 0.051
Chronic disease			
No	1	1	1
Yes	1.71 (1.15–2.54) *p* = 0.008	2.21 (1.41–3.49) *p* = 0.001	1.53 (0.94–2.50) *p* = 0.089
Perpetrate psychological violence			
No	1		1
Yes			23.3 (14.4–37.8) *p* < 0.001
Perpetrate physical violence			
No		1	
Yes		23.3 (14.4–37.8) *p* < 0.001	
Number of close friends			
None	1	1	1
1–2	0.61 (0.13–2.93) *p* = 0.541	0.75 (0.09–6.08) *p* = 0.788	0.36 (0.07–1.73) *p* = 0.356
3 or more	0.55 (0.12–2.58) *p* = 0.451	0.75 (0.09–5.94) *p* = 0.786	0.34 (0.07–1.58) *p* = 0.168
Neighbors’ interest for them			
Interested/very interested	1	1	1
Indifferent	0.59 (0.23–1.49) *p* = 0.263	0.77 (0.28–2.15) *p* = 0.619	0.57 (0.17–1.83) *p* = 0.566
Not interested at all/less interested	1.30 (0.50–3.39) *p* = 0.594	0.82 (0.19–3.46) *p* = 0.785	1.28 (0.39–4.28) *p* = 0.684
Getting the necessary help			
Easy/very easy	1	1	1
Average	0.94 (0.66–1.35) *p* = 0.742	0.98 (0.63–1.53) *p* = 0.932	0.80 (0.51–1.27) *p* = 0.349
Difficult/very difficult	1.61 (1.04–2.49) *p* = 0.0,32	1.77 (1.05–2.97) *p* = 0.032	1.78 (1.07–2.96) *p* = 0.026

## Supplementary Materials

The following are available online at https://www.mdpi.com/1660-4601/16/17/3203/s1, Figure S1: The study framework for analyzing the prevalence and determinants of violence among young people in Serbia age 15–24 years (n = 1448), 2013., Table S1: The characteristics of the youth in the representative sample of the National Health Survey 2013, Serbia, Table S2: The prevalence of various types of violent victimization in the last 12 months by place and response rate, Table S3: Distribution of respondents according to psychological violence victimization in the last 12 months related to their demographic and other characteristics, young people in Serbia age 15–24 years (n = 1448), 2013, Table S4: Distribution of respondents according to physical violence victimization in the last 12 months related to their demographic and other characteristics, young people in Serbia age 15–24 years (n = 1357), 2013, Table S5: Distribution of respondents according to domestic violence victimization in the last 12 months related to their demographic and other characteristics, young people in Serbia age 15–24 years (n = 1567), 2013, Table S6: Distribution of respondents according to violence victimization at school/workplace in the last 12 months related to their demographic and other characteristics, young people in Serbia age 15–24 years (n = 1519), 2013, Table S7: Distribution of respondents according to violence victimization on street in the last 12 months related to their demographic and other characteristics, young people in Serbia age 15–24 years (n = 1519), 2013, Table S8: Distribution of respondents according to violence perpetration in the last 12 months in relation to their demographic and other characteristics, young people in Serbia age 15–24 years (n = 1574), 2013, Table S9: Distribution of respondents according to psychological violence perpetration in the last 12 months according to their demographic and other characteristics, young people in Serbia age 15–24 years (n = 1586), 2013, Table S10: Respondents characteristic according to physical violence perpetration in the last 12 months, young people in Serbia age 15–24 years (n = 1571), 2013, Table S11: Violence victims according to the source of help they have requested from in the last 12 months, young people in Serbia age 15–24 years(n = 1571), 2013, Table S12: Distribution of violence perpetration in the last 12 months, per type of violence, young people in Serbia age 15–24 years (n = 1571), 2013.
