# Supplementary material for: Prevalence and Predictors of Violence Victimization and Violent Behavior among Youths: A Population-Based Study in Serbia
Source: Int J Environ Res Public Health. 2019 Sep 2;16(17):3203. doi: 10.3390/ijerph16173203 (PMC6747162; doi:10.3390/ijerph16173203)
Supplement: Supplementary file 1 [file ijerph-16-03203-s001.pdf]

SUPPLEMENTARY MATERIAL

Figure 1. Study framework for analyzing the prevalence and determinants of violence among young people in Serbia age 15-24 years (n = 1448), 2013.

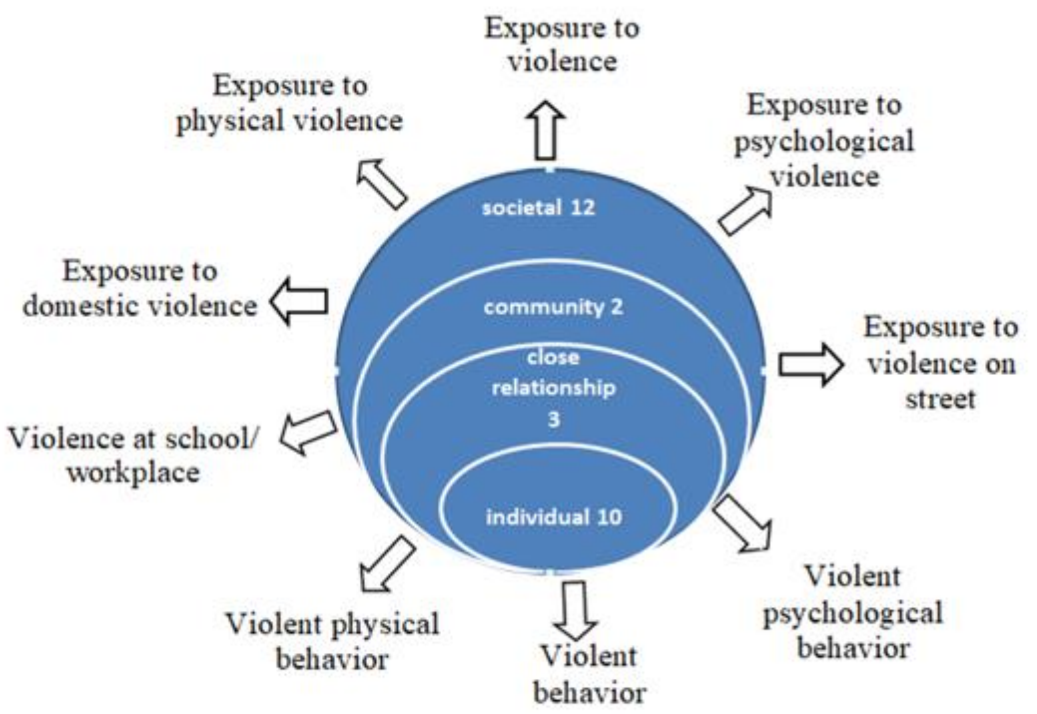

Table S1. The characteristics of the youth in the representative sample of the National Health Survey 2013, Serbia

Characteristics	Respondents	
	n	%
Individual level		
**Sex**		
Male	843	49
Female	879	51
**Age**		
Arithmetic mean ± standard deviation		19.5±2.9
15-19	1031	59.9
20-24	691	40.1
**Employment status**		
Unemployed	1508	87.6
Employed	214	12.4
**Wealth index**		
1st (the lowest)	303	17.6
2nd	352	20.4
3id	337	19.6
4th	357	20.7
5th (the highest)	373	21.7
**Education level**		
Primary or lower	680	39.5
Secondary	963	55.9
Tertiary	79	4.6
**Own health self-perception**		
Very good	1092	63.4
Good	549	31.9
Average	67	3.9
Bad	11	0.6
Very bad	3	0.2
**Chronic disease**		
Yes	125	7.3
No	1597	92.7
**Tobacco smoking**		
Yes	324	74.1
No	113	25.8
Non-response	*1285*	*74.6*
**Binge drinking****		
Yes	568	64.1
No	318	35.9
Non-response	*836*	*48.5*
**Same-sex intercourse**		
Yes	30	3.5
No	833	96.5
Non-response	*859*	*49.9*
**Close-relationship level**		
**Marital status**		
Single	1552	90.1
Married	170	9.9
**Number of close friends to turn to in case of a serious personal problem**		
None	12	0.7
1 or 2	421	14.4
3-5	915	53.1

6 or more	374	21.7
**Number of household members (arithmetic mean ± SD) #**	4.41±1.6 (4.33-4.49); Range: 1–15	
**Community level**		
**Type of settlement**		
Urban	956	55.5
Other settlements	766	44.5
**Region**		
Vojvodina	397	23.1
Belgrade	380	22.1
Sumadija and Western Serbia	517	30.0
Southern and Eastern Serbia	428	24.9
**Societal level**		
**Neighbors' interest in their life, in what is happening to them**		
Very interested	1056	61.3
Interested	552	32.1
Indifferent	73	4.2
Little interested	32	1.9
Not interested	9	0.5
**Getting necessary help from neighbor is**		
Very easy	303	17.6
Easy	649	37.7
Possible	555	32.2
Difficult	174	10.1
Very difficult	41	2.4
**Tracking health information by media**		
TV (yes, occasionally)	979	56.9
No	743	43.1
Internet (yes, occasionally)	939	54.5
No	783	45.5
Newspapers (yes, occasionally)	621	36.1
No	1101	63.9
Radio (yes, occasionally)	327	19
No	1395	81

** Consuming six or more alcoholic beverages in a row.

Table S2. The prevalence of violence victimization in the last 12 months by place and response rate

		Place of various types of violence victimization		
**Type of violence**	**Total**	**In family**	**At school/ workplace**	**On a street**
**Only psychological, n (%)**	94 (5.5%)	5 (2.2%)	**98 (6.4)**	80 (5.2)
Response rate %	*84*	*91*	*89*	*90*
**Only physical, n (%)**	18 (1.3%)	18 (1.1%)	29 (1.9%)	**64 (4.1%)**
Response rate %	*79*	*92*	*89*	*90*
**Combined – Physiological and physical violence, n (%)**	**207 (13.4%)**	44 (2.8%)	106 (7.0%)	**112 (7.3%)**
Response rate %	*90*	*91*	*88*	*89*

Table S3. Distribution of respondents according to psychological violence victimization in the last 12 months related to their demographic and other characteristics, young people in Serbia age 15-24 years (n = 1448), 2013.

Characteristics of respondents	Psychological violence victims				
	No N=1354		Yes N=94		*p**
	n	%	n	%	
**Individual level**					
**Sex**					0.920
Female	641	47.3	44	46.8	
Male	713	52.7	50	53.2	
**Age**					0.509
15-19	803	59.3	59	62.8	
20-24	551	40.7	35	37.2	
**Employment status**	1354		94		0.441
Unemployed	1189	87.8	80	85.1	
Employed	165	12.2	14	14.9	
**Wealth index**					0.303
1st (the lowest)	241	17.8	13	13.8	
2nd	268	19.8	16	17.0	
3id	267	19.7	17	18.1	
4th	300	22.2	20	21.3	
5th the highest)	278	20.5	28	29.8	
**Education level**					0.576
Primary or lower	522	38.6	33	35.1	
Secondary	768	56.7	58	61.7	
Tertiary	64	4.7	3	3.2	
**Psychological violence perpetrator**					**<0.001**
No	1282	96.3	69	75.8	
Yes	49	3.7	22	24.2	
**Physical violence perpetrator**					**<0.001**
No	1274	95.6	81	87.1	
Yes	58	4.4	12	12.9	
**Own health self-perception**					**0.012**
Very good	887	65.5	49	52.1	
Good	413	30.5	35	37.2	
Average	48	3.5	9	9.6	
Bad	5	0.4	1	1.1	
Very bad	1	0.1	0	0.0	
**Chronic disease**					**0.033**
Yes	93	6.9	12	12.8	
No	1261	93.1	82	87.2	
**Tobacco smoking**	402	100	40	100	0.652
Yes	317	78.9	31	77.5	
No	85	21.1	9	22.5	
**Binge drinking****	690	100	68	100	0.214
Yes	424	61.4	47	69.1	
No	266	38.6	21	30.9	
**Same -sex intercourse**					0.295
Yes	20	2.9	3	5.5	
No	667	97.1	52	94.5	
**Close-relationship level**					
**Marital status**					0.919
Single	1220	90.1	85	90.4	
Married	134	9.9	9	9.6	

**Number of close friends (to turn to in case of a serious personal problem)**					**0.007**
None	6	0.4	3	3.2	
1 or 2	313	23.1	25	26.6	
3-5	733	54.1	50	53.2	
6 or more	302	22.3	16	17.0	
**Number of household members** arithmetic mean ± standard deviation (range of arithmetic mean) minimum-maximum		4.45±1.62 (4.36-4.53) (1-15)		4.11±1.57 (3.79-4.43) (1-9)	**0.049#**
**Community level**					
**Societal level**					
**Type of settlement**					**0.023**
Other settlements	610	45.1	31	33.0	
Urban settlement	744	54.9	63	67.0	
**Region**					0.535
Vojvodina	317	23.4	26	27.7	
Belgrade	291	21.5	20	21.3	
Sumadija and Western Serbia	406	30.0	22	23.4	
Southern and Eastern Serbia	340	25.1	26	27.7	
**Neighbors' interest in their life, in what is happening to them**					**0.007**
Very interested	834	61.6	55	58.5	
Interested	434	32.1	28	29.8	
Indifferent	59	4.4	9	9.6	
Little interested	23	1.7	0	0.0	
Not interested	4	0.3	2	2.1	
**Getting the necessary help from neighbor is**					0.221
Very easy	251	18.5	16	17.0	
Easy	516	38.1	26	27.7	
Possible	434	32.1	39	41.5	
Difficult	124	9.2	10	10.6	
Very difficult	29	2.1	3	3.2	
**Tracking health information by media**					
TV (yes/occasionally)	781	58.1	49	52.1	0.262
No	564	41.9	45	47.9	
Internet (yes/occasionally)	755	56.1	59	62.8	0.196
No	591	43.9	35	37.2	
Newspapers (yes/occasionally)	502	37.3	38	40.4	0.536
No	843	62.7	56	59.6	
Radio (yes/occasionally)	267	19.8	16	17.0	0.517
No	1079	80.2	78	83.0	

*Pearson Chi Square test ** Consuming six or more alcoholic beverages in a row. # one-way ANOVA F=3,876 df=1.

Table S4. Distribution of respondents according to physical violence victimization in the last 12 months related to their demographic and other characteristics, young people in Serbia age 15-24 years (n = 1357), 2013.

Characteristics of respondents	Physical violence victims				*p**
	Not victims N=1339		Victims N=18		
	n	%	n	%	
**Individual level**					
**Sex**					
Female	634	47.3	2	11.1	**<0.001**
Male	705	52.7	16	88.9	
**Age**					0.110
15-19	792	59.1	14	77.8	
20-24	547	40.9	4	22.2	
**Employment status**					0.876
Unemployed	1174	87.7	16	88.9	
Employed	165	12.3	2	11.1	
**Wealth index**					**0.019**
1st (the lowest)	237	17.7	0	0.0	
2nd	267	19.9	4	22.2	
3id	264	19.7	3	16.7	
4th	297	22.2	2	11.1	
5th (the highest)	274	20.5	9	50.0	
**Education level**					0.981
Primary or lower	517	38.6	7	38.9	
Secondary	760	56.8	10	55.6	
Tertiary	62	4.6	1	5.6	
**Psychological violence perpetrator**					**0.001**
No	1275	96.7	14	82.4	
Yes	43	3.3	3	17.6	
**Physical violence perpetrator**					**<0.001**
No	1267	95.8	10	55.6	
Yes	56	4.2	8	44.4	
**Own health self-perception**					**0.010**
Very good	877	65.5	9	50.0	
Good	408	30.5	8	44.4	
Average	48	3.6	0	0.0	
Bad	5	0.4	1	5.6	
Very bad	1	0.1	0	0.0	
**Chronic disease**					0.818
Yes	93	6.9	1	5.6	
No	1246	93.1	17	94.4	
**Tobacco smoking**	396	100	6	100	0.421
Yes	312	78.8	6	100.0	
No	84	21.2	0	0.0	
**Binge drinking** **	681	100	16	100	0.175
Yes	418	61.4	13	81.3	
No	263	38.6	3	18.8	
**Same-sex intercourse**					0.365
Yes	20	2.9	1	7.1	
No	658	97.1	13	92.9	
**Close-realtionship level**					
**Marital status**					0.859
Single	1207	90.1	16	88.9	
Married	132	9.9	2	11.1	

**Number of close friends (to turn to in case of a serious personal problem)**					**0.012**
None	6	0.4	1	5.6	
1 or 2	308	23.0	2	11.1	
3-5	724	54.1	12	66.7	
6 or more	301	22.5	3	16.7	
**Number of household members** arithmetic mean ± standard deviation (range of arithmetic mean) minimum-maximum		4.45±1.620 (4.36-4.53) (1-15)		3.89±1.023 (3.38-4.40) (2-6)	0.145#
**Community level**					
**Type of settlement**					0.053
Other settlements	603	45.0	4	22.2	
Urban settlement	736	55.0	14	77.8	
**Region**					0.657
Vojvodina	314	23.5	5	27.8	
Belgrade	287	21.4	5	27.8	
Sumadija and Western Serbia	404	30.2	3	16.7	
Southern and Eastern Serbia	334	24.9	5	27.8	
**Societal level**					
**Neighbors' interest in their life, in what is happening to them**					0.819
Very interested	828	61.8	13	72.2	
Interested	426	31.8	5	27.8	
Indifferent	59	4.4	0	0.0	
Little interested	22	1.6	0	0.0	
Not interested	4	0.3	0	0.0	
**Getting the necessary help from neighbor is**					0.087
Very easy	246	18.4	2	11.1	
Easy	514	38.4	3	16.7	
Possible	427	31.9	9	50.0	
Difficult	123	9.2	4	22.2	
Very difficult	29	2.2	0	0.0	
**Tracking health information by media**					
TV (yes/occasionally)	771	57.9	10	58.8	0.926
No	561	42.1	7	41.2	
Internet (yes/occasionally)	746	56.0	12	70.6	0.274
No	586	44.0	5	29.4	
Newspapers (yes/occasionally)	495	37.2	7	41.2	0.680
No	836	62.8	10	58.8	
Radio (yes/occasionally)	263	19.7	2	11.8	0.705
No	1069	80.3	15	88.2	

*Pearson Chi Square test ** Consuming six or more alcoholic beverages in a row. # one-way ANOVA F = 2,121; df=1.

Table S5. Distribution of respondents according to domestic violence victimization in the last 12 months related to their demographic and other characteristics, young people in Serbia age 15-24 years (n = 1567), 2013.

Characteristics of respondents	Domestic violence victims				*p**
	Not victims N=1523		Victims N=44		
	n	%	n	%	
**Individual level**					
**Sex**					0.827
Female	736	48.3	22	50.0	
Male	787	51.7	22	50.0	
**Age**					0.442
15-19	902	59.2	23	52.3	
20-24	621	40.8	21	47.7	
**Employment status**					0.816
Unemployed	1332	87.5	39	88.6	
Employed	191	12.5	5	11.4	
**Wealth index**					0.287
1st (the lowest)	265	17.4	13	29.5	
2nd	307	20.2	7	15.9	
3id	303	19.9	6	13.6	
4th	322	21.1	8	18.2	
5th (the highest)	326	21.4	10	22.7	
**Education level**					0.452
Primary or lower	586	38.5	20	45.5	
Secondary	866	56.9	21	47.7	
Tertiary	71	4.7	3	6.8	
**Psychological violence perpetrators**					**<0.001**
No	1416	94.8	27	65.9	
Yes	78	5.2	14	34.1	
**Physical violence perpetrators**					**<0.001**
No	1396	94.0	29	70.7	
Yes	89	6.0	12	29.3	
**Own health self-perception**					**<0.001**
Very good	978	64.2	21	47.7	
Good	479	31.5	15	34.1	
Average	55	3.6	5	11.4	
Bad	9	0.6	2	4.5	
Very bad	2	0.1	1	2.3	
**Chronic disease**					**0.006**
Yes	109	7.2	8	18.2	
No	1414	92.8	36	81.8	
**Tobacco smoking**	469		20		0.393
Yes	370	78.9	16	80.0	
No	99	21.1	4	20.0	
**Binge drinking** **	794		30		0.566
Yes	500	63.0	19	63.3	
No	294	37.0	11	36.7	
**Same-sex intercourse**					**<0.001**
Yes	78	5.2	14	34.1	
No	1416	94.8	27	65.9	
**Close-relationship level**					
**Marital status**					0.218
Single	1368	89.8	37	84.1	
Married	155	10.2	7	15.9	

**Number of close friends (to turn to in case of a serious personal problem)**					**<0.001**
None	9	0.6	2	4.5	
1 or 2	358	23.5	18	40.9	
3-5	825	54.2	13	29.5	
6 or more	331	21.7	11	25.0	
**Number of household members** arithmetic mean ± standard deviation (range of arithmetic mean) minimum-maximum		4.41±1.601 (4.33-4.49) (1-15)		4.11±1.498 (3.66-4.57) (1-8)	0.203#
**Community level**					
**Type of settlement**					0.096
Other settlements	677	44.5	14	31.8	
Urban settlement	846	55.5	30	68.2	
**Region**					0.683
Vojvodina	356	23.4	11	25.0	
Belgrade	327	21.5	9	20.5	
Sumadija and Western Serbia	454	29.8	10	22.7	
Southern and Eastern Serbia	386	25.3	14	31.8	
**Societal level**					
**Neighbors' interest in their life, in what is happening to them**					**0.004**
Very interested	941	61.8	22	50.0	
Interested	485	31.8	13	29.5	
Indifferent	67	4.4	5	11.4	
Little interested	23	1.5	3	6.8	
Not interested	7	0.5	1	2.3	
**Getting the necessary help from neighbor is**					0.375
Very easy	268	17.6	6	13.6	
Easy	571	37.5	13	29.5	
Possible	497	32.6	16	36.4	
Difficult	150	9.8	8	18.2	
Very difficult	37	2.4	1	2.3	
**Tracking health information by media**					
TV (yes/occasionally)	869	57.4	28	63.6	0.429
No	645	42.6	16	36.4	
Internet (yes/occasionally)	844	55.7	26	59.1	0.403
No	670	44.3	18	40.9	
Newspapers (yes/occasionally)	554	36.6	20	45.5	0.232
No	959	63.4	24	54.5	
Radio (yes/occasionally)	293	19.4	12	27.3	0.214
No	1221	80.6	32	72.7	

*Pearson Chi Square test ** Consuming six or more alcoholic beverages in a row. # one-way ANOVA F=1.467 df=1.

Table S6. Distribution of respondents according to violence victimization at school/workplace in the last 12 months related to their demographic and other characteristics, young people in Serbia age 15-24 years (n = 1519), 2013.

Characteristics of respondents	Violence victims at school/workplace				p*
	No N=1413		Yes N=106		
	n	%	n	%	
**Individual level**					
**Sex**					0.134
Female	733	51.9	59	55.7	
Male	680	48.1	47	44.3	
**Age**					**0.003**
15-19	834	59.0	78	73.6	
20-24	579	41.0	28	26.4	
**Employment status**					0.455
Unemployed	1239	87.7	94	88.7	
Employed	174	12.3	12	11.3	
**Wealth index**					0.302
1st (the lowest)	252	17.8	14	13.2	
2nd	276	19.5	25	23.6	
3id	275	19.5	22	20.8	
4th	309	21.9	17	16.0	
5th (the highest)	301	21.3	28	26.4	
**Education level**					**0.006**
Primary or lower	539	38.5	57	53.8	
Secondary	809	56.9	45	42.5	
Tertiary	65	4.7	4	3.8	
**Psychological violence perpetrators**					**<0.001**
No	1331	94.8	63	63.6	
Yes	59	5.2	36	36.4	
**Physical violence perpetrators**					**<0.001**
No	1325	94.0	70	68.6	
Yes	72	6.0	32	31.4	
**Own health self-perception**					**<0.001**
Very good	917	64.2	52	49.1	
Good	430	31.5	49	46.2	
Average	57	3.6	2	1.9	
Bad	7	0.6	3	2.8	
Very bad	2	0.1	0	0.0	
**Chronic disease**					0.054
Yes	101	7.1	13	12.3	
No	1312	92.9	93	87.7	
**Tobacco smoking**	342	100	40	100	0.677
Yes	251	73.4	31	77.5	
No	91	26.6	9	22.5	
**Binge drinking** **	739	100	74	100	0.545
Yes	463	62.7	49	66.2	
No	276	37.3	25	33.8	
**Same-sex intercourse**					0.698
Yes	24	3.3	2	4.3	
No	706	96.7	44	95.7	
**Close-relationship level**					
**Marital status**					0.276

Single	1274	90.2	99	93.4	
Married	139	9.8	7	6.6	
**Number of close friends (to turn to in case of a serious personal problem)**					0.223
None	10	0.7	0	0.0	
1 or 2	329	23.3	33	31.1	
3-5	761	53.9	49	46.2	
6 or more	313	22.2	24	22.6	
**Number of household members** arithmetic mean ± standard deviation (range of arithmetic mean) minimum-maximum		4.42±1.611 (4.34-4.51) (1-15)		4.18±1.517 (3.89-4.47) (1-9)	0.112#
**Community level**					
**Type of settlement**					0.586
Other settlements	788	55.8	62	58.5	
Urban settlement	625	44.2	44	41.5	
**Region**					0.561
Vojvodina	329	23.3	26	24.5	
Belgrade	309	21.9	17	16.0	
Sumadija and Western Serbia	417	29.5	33	31.1	
Southern and Eastern Serbia	358	25.3	30	28.3	
**Societal level**					
**Neighbors' interest in their life, in what is happening to them**					0.946
Very interested	866	61.3	65	61.3	
Interested	454	32.1	35	33.0	
Indifferent	64	4.5	4	3.8	
Little interested	22	1.6	2	1.9	
Not interested	7	0.5	0	0.0	
**Getting necessary help from neighbor is**					0.698
Very easy	252	17.8	19	17.9	
Easy	535	37.9	35	33.0	
Possible	457	32.3	38	35.8	
Difficult	139	9.8	10	9.4	
Very difficult	30	2.1	4	3.8	
**Tracking health information by media**					
TV (yes/occasionally)	816	58.1	53	50.5	0.311
No	589	41.9	52	49.5	
Internet (yes/occasionally)	791	56.3	58	55.2	0.978
No	614	43.7	47	44.8	
Newspapers (yes/occasionally)	529	37.7	34	32.4	0.547
No	875	62.3	71	67.6	
Radio (yes/occasionally)	282	20.1	14	13.3	0.213
No	1123	79.9	91	86.7	

*Pearson Chi Square test, ** Consuming six or more alcoholic beverages in a row. # one-way ANOVA F=2,307; df=1.

Table S7. Distribution of respondents according to violence victimization on the street in the last 12 months related to their demographic and other characteristics, young people in Serbia age 15-24 years (n = 1519), 2013.

Characteristics of respondents	Violence victims on the street				*p**
	No N=1427		Yes N=112		
	n	%	n	%	
**Individual level**					
**Sex**					**<0.001**
Female	673	47.2	33	29.5	
Male	754	52.8	79	70.5	
**Age**					0.718
15-19	853	59.8	65	58.0	
20-24	574	40.2	47	42.0	
**Employment status**					0.941
Unemployed	1252	87.7	98	87.5	
Employed	175	12.3	14	12.5	
**Wealth index**					**0.005**
1st (the lowest)	249	17.4	17	15.2	
2nd	287	20.1	19	17.0	
3id	283	19.8	20	17.9	
4th	313	21.9	16	14.3	
5th (the highest)	295	20.7	40	35.7	
**Education level**					0.459
Primary or lower	556	39.0	37	33.0	
Secondary	805	56.4	69	61.6	
Tertiary	66	4.6	6	5.4	
**Psychological violence perpetrators**					**<0.001**
No	1343	95.8	61	58.7	
Yes	59	4.2	43	41.3	
**Physical violence perpetrators**					**<0.001**
No	1337	95.0	66	65.3	
Yes	71	5.0	35	34.7	
**Own health self-perception**					**<0.001**
Very good	919	64.4	65	58.0	
Good	447	31.3	34	30.4	
Average	53	3.7	8	7.1	
Bad	7	0.5	4	3.6	
Very bad	1	0.1	1	0.9	
**Chronic disease**					0.342
Yes	105	7.4	11	9.8	
No	1322	92.6	101	90.2	
**Tobacco smoking**	426	100	59	100	0.166
Yes	333	78.2	52	88.1	
No	93	21.8	7	11.9	
**Binge drinking** **	738	100	89	100	0.004
Yes	456	61.8	69	77.5	
No	282	38.2	20	22.5	
**Same-sex intercourse**	720	100	81	100	0.166
Yes	23	3.2	5	6.2	
No	697	96.8	76	93.8	
**Close-relatioship level**					
**Marital status**					0.701
Single	1290	90.4	100	89.3	
Married	137	9.6	12	10.7	

**Number of close friends (to turn to in case of a serious personal problem)**					**0.015**
None	7	0.5	3	2.7	
1 or 2	334	23.4	33	29.5	
3-5	767	53.7	56	50.0	
6 or more	319	22.4	20	17.9	
**Number of household members** arithmetic mean ± standard deviation (range of arithmetic mean) minimum-maximum		4.43±1.613 (4.35-4.51) (1-15)		4.09±1.430 (3.82-4.36) (1-8)	0.128#
**Community level**					
**Type of settlement**					**<0.001**
Other settlements	642	45.0	30	26.8	
Urban settlement	785	55.0	82	73.2	
**Region**					0.374
Vojvodina	334	23.4	25	22.3	
Belgrade	304	21.3	29	25.9	
Sumadija and Western Serbia	428	30.0	26	23.2	
Southern and Eastern Serbia	361	25.3	32	28.6	
**Societal level**					
**Neighbors' interest in their life, in what is happening to them**					**<0.001**
Very interested	888	62.2	54	48.2	
Interested	449	31.5	48	42.9	
Indifferent	64	4.5	4	3.6	
Little interested	22	1.5	3	2.7	
Not interested	4	0.3	3	2.7	
**Getting necessary help from neighbor is**					**<0.001**
Very easy	258	18.1	14	12.5	
Easy	544	38.1	34	30.4	
Possible	464	32.5	36	32.1	
Difficult	131	9.2	21	18.8	
Very difficult	30	2.1	7	6.3	
**Tracking health information by media**					
TV (yes/occasionally)	818	57.6	66	59.5	0.832
No	602	42.4	45	40.5	
Internet (yes/occasionally)	279	19.6	24	21.6	0.712
No	1141	80.4	87	78.4	
Newspapers (yes/occasionally)	528	37.2	40	36.0	0.824
No	891	62.8	71	64.0	
Radio (yes/occasionally)	797	56.1	66	59.5	0.214
No	623	43.9	45	40.5	

*Pearson Chi Square test , ** Consuming six or more alcoholic beverages in a row. # one-way ANOVA F=4,714; df=1.

Table S8. Distribution of respondents according to violence perpetration in the last 12 months in relation to their demographic and other characteristics, young people in Serbia age 15-24 years (n = 1574), 2013.

Characteristics of respondents	Violence perpetrators				p*
	No (N=1397)		Yes (N=177)		
	n	%	n	%	
**Individual level**					
**Sex**					**<0.001**
Female	752	53.8	55	31.1	
Male	645	46.2	122	68.9	
**Age**					0.289
15-19	826	59.1	112	63.3	
20-24	571	40.9	65	36.7	
**Employment status**					0.061
Unemployed	1218	87.2	163	92.1	
Employed	179	12.8	14	7.9	
**Wealth index**					0.080
1st (the lowest)	251	18.0	29	16.4	
2nd	285	20.4	32	18.1	
3id	277	19.8	29	16.4	
4th	297	21.3	34	19.2	
5th (the highest)	287	20.5	53	29.9	
**Education level**					0.720
Primary or lower	540	38.7	74	41.8	
Secondary	791	56.6	95	53.7	
Tertiary	66	4.7	8	4.5	
**Own health self-perception**					**<0.001**
Very good	901	64.5	91	51.4	
Good	436	31.2	70	39.5	
Average	51	3.7	11	6.2	
Bad	8	0.6	3	1.7	
Very bad	1	0.1	2	1.1	
**Chronic disease**					0.451
Yes	104	7.4	16	9.0	
No	1293	92.6	161	91.0	
**Tobacco smoking**					0.359
Yes	329	79.1	67	82.7	
No	87	20.9	14	17.3	
**Binge drinking** **					<0.001
Yes	429	60.5	107	79.9	
No	280	39.5	27	20.1	
**Same-sex intercourse**					0.001
Yes	18	2.6	10	9.0	
No	686	97.4	101	91.0	
**Close-relationship level**					
**Marital status**					**0.006**
Single	1251	89.5	170	96.0	
Married	146	10.5	7	4.0	
**Number of close friends (to turn to in case of a serious personal problem)**					0.832
None	9	0.6	2	1.1	
1 or 2	337	24.1	46	26.0	
3-5	743	53.2	91	51.4	
6 or more	308	22.0	38	21.5	

**Number of household members** arithmetic mean ± standard deviation (range of arithmetic mean) minimum-maximum		4.42±1.610 (4.34-4.51) (1-15)		4.18±1.553 (3.95-4.41) (1-12)	**0.042#**
**Community level**					
**Type of settlement**					0.115
Other settlements	624	44.7	68	38.4	
Urban settlement	773	55.3	109	61.6	
**Region**					0.553
Vojvodina	330	23.6	36	20.3	
Belgrade	299	21.4	42	23.7	
Sumadija and Western Srrbia	412	29.5	48	27.1	
Southern and Eastern Serbia	356	25.5	51	28.8	
**Societal level**					
**Neighbors' interest in their life, in what is happening to them**					0.761
Very interested	857	61.3	107	60.5	
Interested	444	31.8	60	33.9	
Indifferent	66	4.7	5	2.8	
Little interested	25	1.8	4	2.3	
Not interested	5	0.4	1	0.6	
**Getting the necessary help from neighbor is**					0.142
Very easy	251	18.0	29	17.9	
Easy	522	37.4	64	33.0	
Possible	459	32.9	52	35.8	
Difficult	135	9.7	24	9.4	
Very difficult	30	2.1	8	3.8	
**Tracking health information by media**					
TV (Yes/occasionally)	819	59.0	88	50.5	0.068
No	569	41.0	87	49.5	
Internet (Yes/occasionally)	772	55.6	102	55.2	0.773
No	617	44.4	73	44.8	
Newspapers (Yes/occasionally)	525	37.8	55	32.4	0.205
No	863	62.2	120	67.6	
Radio (Yes/occasionally)	278	20.0	30	13.3	0.464
No	1111	80.0	145	86.7	

*Pearson Chi Square test , ** Consuming six or more alcoholic beverages in a row. # one-way ANOVA F=3,565; df=1.

Table S9. Distribution of respondents according to only psychological violence perpetration in the last 12 months according to their demographic and other characteristics, young people in Serbia age 15-24 years (n = 1586), 2013.

Characteristics of respondents	Psychological violence perpetrators				p*
	No (N=1475)		Yes (N=111)		
	n	%	n	%	
**Individual level**					
**Sex**					**0.005**
Female	777	52.7	43	38.7	
Male	698	47.3	68	61.3	
**Age**					0.224
15-19	870	59.0	72	64.9	
20-24	605	41.0	39	35.1	
**Employment status**					0.087
Unemployed	1287	87.3	103	92.8	
Employed	188	12.7	8	7.2	
**Wealth index**					**0.038**
1st (the lowest)	259	17.6	18	16.2	
2nd	302	20.5	19	17.1	
3id	296	20.1	16	14.4	
4th	312	21.2	21	18.9	
5th (the highest)	306	20.7	37	33.3	
**Education level**					0.137
Primary or lower	564	38.2	53	47.7	
Secondary	840	56.9	53	47.7	
Tertiary	71	4.8	5	4.5	
**Physical violence perpetrator**					**<0.001**
No	1397	96.3	52	53.1	
Yes	53	3.7	46	46.9	
**Own health self-perception**					**<0.001**
Very good	947	64.2	56	50.5	
Good	466	31.6	42	37.8	
Average	53	3.6	8	7.2	
Bad	8	0.5	3	2.7	
Very bad	1	0.1	2	1.8	
**Chronic disease**					**0.037**
Yes	106	7.2	14	12.6	
No	1369	92.8	97	87.4	
**Tobacco smoking**	454	100	50	100	0.141
Yes	358	78.9	44	88.0	
No	96	21.1	6	12.0	
**Binge drinking** **	760	100	83	100	**0.025**
Yes	473	62.2	62	74.7	
No	287	37.8	21	25.3	
**Same-sex intercourse**	761	100	61	100	**0.020**
Yes	21	2.8	5	8.2	
No	740	97.2	56	91.8	
**Close-relationship level**					
**Marital status**					**0.046**
Single	1322	89.6	106	95.5	
Married	153	10.4	5	4.5	
**Number of close friends (to turn to in case of a serious personal problem)**					0.985
None	10	0.7	1	0.9	

1 or 2	360	24.4	27	24.3	
3-5	783	53.1	60	54.1	
6 or more	322	21.8	23	20.7	
**Number of household members** arithmetic mean ± standard deviation (range of arithmetic mean) minimum-maximum		4.44±1.629 (4.36-4.52) (1-15)		3.86±1.022 (3.67-4.06) (1-7)	**<0.001#**
**Community level**					
**Type of settlement**					0.097
Other settlements	651	44.1	40	36.0	
Urban settlement	824	55.9	71	64.0	
**Region**					0.927
Vojvodina	344	23.3	25	22.5	
Belgrade	319	21.6	26	23.4	
Sumadija and Western Serbia	434	29.4	30	27.0	
Southern and Eastern Serbia	378	25.6	30	27.0	
**Societal level**					
**Neighbors' interest in their life, in what is happening to them**					0.892
Very interested	902	61.2	66	59.5	
Interested	473	32.1	39	35.1	
Indifferent	68	4.6	4	3.6	
Little interested	25	1.7	2	1.8	
Not interested	7	0.5	0	0.0	
**Getting the necessary help from neighbor is**					0.184
Very easy	261	17.7	19	17.1	
Easy	548	37.2	37	33.3	
Possible	486	32.9	33	29.7	
Difficult	144	9.8	19	17.1	
Very difficult	36	2.4	3	2.7	
**Tracking health information by media**					
TV (yes/occasionally)	853	58.2	60	54.5	0.733
No	612	41.8	50	45.5	
Internet (yes/occasionally)	812	55.4	65	59.1	0.408
No	654	44.6	45	40.9	
Newspapers (yes/occasionally)	544	37.1	39	35.5	0.914
No	921	62.9	71	64.5	
Radio (yes/occasionally)	287	19.6	14	14.0	0.631
No	1179	80.4	86	86.0	

*Pearson Chi Square test, ** Consuming six or more alcoholic beverages in a row. # one-way ANOVA F=13.471; df=1.

Table S10. Respondents characteristic according to physical violence perpetration in the last 12 months, young people in Serbia age 15-24 years (n = 1571), 2013.

Characteristics of respondents	Physically violent behavior				p*
	No (N=1459)		Yes (N=112)		
	n	%	n	%	
**Individual level**					
**Sex**					**<0.001**
Female	781	53.5	21	18.8	
Male	678	46.5	91	81.3	
**Age**					0.811
15-19	869	59.6	69	61.1	
20-24	590	40.4	44	38.9	
**Employment status**					0.399
Unemployed	1276	87.5	101	90.2	
Employed	183	12.5	11	9.8	
**Wealth index**					0.134
1st (the lowest)	261	17.9	18	15.9	
2nd	297	20.4	19	16.8	
3id	284	19.5	22	19.5	
4th	311	21.3	19	16.8	
5th (the highest)	306	21.0	35	31.0	
**Education level**					0.757
Primary or lower	571	39.1	40	35.7	
Secondary	820	56.2	66	58.9	
Tertiary	68	4.7	6	5.4	
**Psychological violence perpetrator**					**<0.001**
No	1397	96.4	53	53.5	
Yes	52	3.6	46	46.5	
**Own health self-perception**					0.118
Very good	933	63.9	63	56.3	
Good	458	31.4	42	37.5	
Average	57	3.9	4	3.6	
Bad	9	0.6	2	1.8	
Very bad	2	0.1	1	0.9	
**Chronic disease**					0.878
Yes	110	7.5	8	7.1	
No	1349	92.5	104	92.9	
**Tobacco smoking**	440	100	55	100	0.928
Yes	349	79.3	43	78.2	
No	91	20.7	12	21.8	
**Binge drinking** **	750	100	91	100	**<0.001**
Yes	460	61.3	75	82.4	
No	290	38.7	16	17.6	
**Same-sex intercourse**	733	100	79	100	0.117
Yes	22	3.0	5	6.3	
No	711	97.0	74	93.7	
**Close-relationship level**					
**Marital status**					**0.023**
Single	1311	89.9	108	96.4	
Married	148	10.1	4	3.6	
**Number of close friends (to turn to in case of a serious personal problem)**					0.551
None	9	0.6	2	1.8	
1 or 2	354	24.3	28	25.0	
3-5	774	53.1	58	51.8	

6 or more	322	22.1	24	21.4	
**Number of household members** arithmetic mean ± standard deviation (range of arithmetic mean) minimum-maximum		4.39±1.596 (4.31-4.47) (1-15)		4.43±1.707 (4.11-4.75) (1-12)	0.820#
**Community level**					
**Type of settlement**					0.416
Other settlements	644	44.1	45	40.2	
Urban settlement	815	55.9	67	59.8	
**Region**					0.262
Vojvodina	347	23.8	20	17.9	
Belgrade	312	21.4	28	25.0	
Sumadija and Western Serbia	429	29.4	29	25.9	
Southern and Eastern Serbia	371	25.4	35	31.3	
**Societal level**					
**Neighbors' interest in their life, in what is happening to them**					0.671
Very interested	887	60.8	73	65.2	
Interested	474	32.5	33	29.5	
Indifferent	68	4.7	3	2.7	
Little interested	25	1.7	2	1.8	
Not interested	5	0.3	1	0.9	
**Getting necessary help from neighbor is**					**0.007**
Very easy	259	17.8	20	17.9	
Easy	544	37.3	40	35.7	
Possible	483	33.1	29	25.9	
Difficult	143	9.8	15	13.4	
Very difficult	30	2.1	8	7.1	
**Tracking health information by media**					
TV (yes/occasionally)	843	58.6	55	49.5	0.103
No	596	41.4	56	50.5	
Internet (yes/occasionally)	808	55.7	67	60.4	0.261
No	642	44.3	44	39.6	
Newspapers (yes/occasionally)	549	37.9	21	20.8	0.063
No	900	62.1	80	79.2	
Radio (yes/occasionally)	289	19.9	18	16.2	0.255
No	1161	80.1	93	83.8	

*Pearson Chi Square test, ** Consuming six or more alcoholic beverages in a row. # one-way ANOVA F=0,052; df=1.

Table S11. Violence victimization according to the source of help they have requested from in the last 12 months, young people in Serbia age 15–24 years(n = 1571), 2013.

	Type and place of violence victimization					
**Requested help from n (%)**	**Physical & psychological n=207(100%)**	**Only psychological n=94(100%)**	**Only physical n=18(100%)**	**At home n=44(100%)**	**At school / workplace n=106(100%)**	**On street n=112(100%)**
Family, friends	72 (41%)	31 (36%)	6 (35%)	16 (41%)	41 (44%)	41 (45%)
Police	25 (15%)	3 (4%)	2 (12%)	8 (26%)	10 (11%)	15 (16%)
Teacher, professor	25 (15%)	9 (11%)	1 (6%)	7 (19%)	20 (21%)	10 (11%)
Social worker	10 (6%)	3 (3%)	0	6 (15%)	5 (6%)	5 (5%)
Medical worker	8 (5%)	2 (2%)	0	5 (13%)	5 (6%)	4 (4%)
SOS service	6 (4%)	2 (2%)	0	2 (6%)	4 (4%)	4 (4%)
Response Rates	***(80-84%)***	***(90-91%)***	***(94%)***	***(71-87%)***	***(85-90%)***	***(80-84%)***

Table S12. Distribution of violence perpetrators in the last 12 months, per type of violence, young people in Serbia age 15-24 years (n = 1571), 2013.

**Type of violence perpetration**	**N (%)**
Psychological (n= 1586)	111 (7.0%)
Response rate	*92.1%*
Physical (n= 1571)	112 (7.1%)
Response rate	*91.8%*
Psychological and physical (n= 1574)	177 (10.3)
Response rate	*91.4%*
